# Inhibitory Effects of Daiokanzoto (Da-Huang-Gan-Cao-Tang) on P-Glycoprotein

**DOI:** 10.1155/2012/361516

**Published:** 2012-08-27

**Authors:** Yuka Watanabe, Nobutomo Ikarashi, Toshiyuki Satoh, Kiyomi Ito, Wataru Ochiai, Kiyoshi Sugiyama

**Affiliations:** Department of Clinical Pharmacokinetics, Hoshi University, 2-4-41 Ebara, Shinagawa-ku, Tokyo 142-8501, Japan

## Abstract

We have studied the effects of various Kampo medicines on P-glycoprotein (P-gp), a drug transporter, *in vitro*. The present study focused on Daiokanzoto (Da-Huang-Gan-Cao-Tang), which shows the most potent inhibitory effects on P-gp among the 50 Kampo medicines studied, and investigated the P-gp inhibitory effects of Daiokanzoto herbal ingredients (rhubarb and licorice root) and their components by an ATPase assay using human P-gp membrane. Both rhubarb and licorice root significantly inhibited ATPase activity, and the effects of rhubarb were more potent than those of licorice root. The content of rhubarb in Daiokanzoto is double that in licorice root, and the inhibition patterns of Daiokanzoto and rhubarb involve both competitive and noncompetitive inhibition, suggesting that the inhibitory effects of Daiokanzoto are mainly due to rhubarb. Concerning the components of rhubarb, concentration-dependent inhibitory effects were observed for (−)-catechin gallate, (−)-epicatechin gallate, and (−)-epigallocatechin gallate. In conclusion, rhubarb may cause changes in the drug dispositions of P-gp substrates through the inhibition of P-gp. It appears that attention should be given to the interactions between these drugs and Kampo medicines containing rhubarb as an herbal ingredient.

## 1. Introduction

P-glycoprotein (P-gp), a member of the ATP-binding cassette (ABC) transporter family, has a molecular weight of 170 kDa and is a glycoprotein consisting of 1,280 amino acids. P-gp was discovered in 1986 as a product of the MDRI gene that causes multidrug resistance in cancer cells [1]. P-gp contains 12 transmembrane domains and two ATP binding sites [2, 3]. When P-gp recognizes a substrate, ATP binds to ATP binding sites. Next, ATPase activates and hydrolyzes ATP. P-gp utilizes the energy of ATP hydrolysis to transport various substrates across cellular membranes. P-gp is expressed in both cancer cells and normal tissue and is involved in the absorption, distribution, and excretion of drugs (anticancer drug and so on) and toxic substances in gastrointestinal epithelial cells, proximal renal tubule epithelial cells, hepatocyte canalicular membrane, cerebral capillary endothelial cells, and placental trophoblast cells [4–6]. Therefore, the drug which inhibits P-gp activity affects pharmacokinetics of P-gp substrates and resistance of multiple anticancer drugs.

The Ministry of Health, Labor and Welfare currently requires data on interactions (as appended documentation) to be included in applications for new drug approvals. Consequently, the mechanisms of many interactions have also been clarified. However, most research on drug-drug interactions involves Western drugs, and because drug interactions are generally considered unlikely to occur, few clinical reports on pharmacokinetic changes or the onset of adverse events due to combined use have been published for herbal and Kampo medicines. At present, examples of the effects of herbal and Kampo medicines on pharmacokinetics include the inhibition of cytochrome P450 (CYP) 1A2, 2C9, and 3A4, which are drug-metabolizing enzymes (reported by Saireito (Chai-Ling-Tang)) [7], the inhibition of CYP2C9 and 3A4 (reported by Hochuekkito (Bu-Zhong-Yi-Qi-Tang)) [7], and the inhibition of CYP3A4 (reported by Shosaikoto (Xiao-Chai-Hu-Tang)) [7]. Furthermore, many herbal medicines have been shown to inhibit CYP2D6 and CYP3A4 *in vitro *[8, 9]. It has also been reported that carbon tetrachloride-induced liver damage can be prevented by inhibition of the expression and activity of CYP2E1 by glycyrrhetic acid, a component of licorice root [10]; likewise, baicalein and wogonin (components of *Scutellaria *root) cause the induction and inhibition of CYP1A2, and baicalein causes inhibition of CYP3A4 [11, 12]. In an *in vitro* study of interactions involving P-gp using P-gp-expressing cells, Shosaikoto was reported to inhibit the efflux of digoxin from cells [13]. However, little research has examined interactions of Kampo medicines in comparison to those of Western medicines. In particular, no systematic research has focused on P-gp-mediated interactions. 

Our review of screenings by ATPase assay to date revealed that many of the 50 Kampo medicines we have examined can inhibit P-gp [14]. Because these Kampo medicines comprise multiple herbal ingredients, it is possible that P-gp inhibitory effects are caused by these medicine's common herbal ingredient(s). Therefore, we focused on Daiokanzoto (Da-Huang-Gan-Cao-Tang), which exhibited the strongest inhibitory effects (during screening). Daiokanzoto is a Kampo medicine (which is covered by health insurance) that treats constipation. It is formed by combining two herbal medicines, rhubarb (*Rheum palmatum* Linné) and licorice root (*Glycyrrhiza uralensis* Fischer), in a ratio of 2 : 1. The present study investigated the P-gp inhibitory effects of the herbal ingredients rhubarb and licorice root and their components.

## 2. Materials and Methods

### 2.1. Materials

Human P-gp membranes were purchased from BD Gentest (Woburn, MA, USA). Rhubarb (Lot; 50728-1) and licorice root (Lot; 081101) were purchased from Takasago Yakugyo Co., Ltd. (Osaka, Japan) and Yamamoto Yakuhin Kogyo Co., Ltd. (Tokyo, Japan), respectively. Adenosine-5′-triphosphate magnesium salt (MgATP), aloe emodin, (+)-catechin, emodin, gallic acid, malachite green, ouabain, rhein, sodium molybdate dehydrate, sodium orthovanadate, trichloroacetic acid, (±)-verapamil hydrochloride, and vinblastine were purchased from Sigma-Aldrich, Inc. (St. Louis, MO). (−)-catechin, (−)-catechin gallate, digoxin, (−)-epicatechin, (−)-epicatechin gallate, (−)-epigallocatechin, (−)-epigallocatechin gallate, ketoconazole, sennoside A, sennoside B, and sodium azide were purchased from Wako Pure Chemical Industries, Ltd. (Osaka, Japan). All other reagents were of the highest commercially available grade.

### 2.2. Lyophilized Extracts of Daiokanzoto and Its Herbal Ingredients

Daiokanzoto was prepared by weighing and mixing 20 g of rhubarb and 10 g of licorice root, adding 300 mL of purified water, and extracting (by heating) until the liquid volume was reduced by half. The mixture was cooled to room temperature, and the filtrate obtained by natural filtration was lyophilized overnight in a lyophilizer. With this process, 4.6 g of lyophilized extract of Daiokanzoto was obtained with an extraction rate of 15.3%.

For the herbal ingredients, 20 g of rhubarb and 24 g of licorice root were weighed, and each was extracted and lyophilized using the same method as used for Daiokanzoto. Weights of 4.9 g and 4.5 g of lyophilized extracts of rhubarb and licorice root, respectively, were obtained at extraction rates of 20.6% and 18.9%.

These lyophilized extracts were dissolved in purified water prior to use in this study.

### 2.3. ATPase Assay

The ATPase activity of human P-gp membranes was determined by measuring inorganic phosphate liberation according to the procedure reported by Sarkadi et al. (with some modifications) [15, 16]. The human P-gp membranes (2 *μ*g of protein) were suspended in 10 *μ*L of the incubation medium, which contained 50 mM Tris-Mes (pH 6.8), 2 mM dithiothreitol, 50 mM KCl, 2 mM EGTA, 2 mM ouabain, and 5 mM sodium azide. This medium was mixed in a 96-well plate with 10 *μ*L of a test compound solution and 10 *μ*L of distilled water or 250 *μ*M verapamil. The ATPase reaction was started by adding 20 *μ*L of 4 mM MgATP solution to the reaction mixture (30 *μ*L), and the incubation was maintained at 37°C for 30 min. The reaction was stopped by the addition of 20 *μ*L of 5% trichloroacetic acid.

The amount of liberated inorganic phosphate was measured by a method described by Carter and Karl [17]. Briefly, 42 *μ*L of solution A (2 M HCl : 0.1 M sodium molybdate = 4 : 3), 18 *μ*L of solution B (0.042% (w/v) malachite green solution in 1% (w/v) polyvinyl alcohol) and 120 *μ*L of solution C (7.8% (v/v) sulfuric acid) were added, and the mixture was allowed to stand at room temperature for 1 h, after which the absorbance was measured at a wavelength of 630 nm using a microplate reader. The ATPase activity was estimated by the difference in the phosphate levels between 0 min (reaction stopped immediately) and 30 min incubation periods. The background phosphate levels in the test solutions were measured after incubation without MgATP and were found to be much lower than the amounts liberated in the ATPase reactions.

### 2.4. Inhibitory Effects on ATPase Activity in the Presence of Various P-gp Substrates

Daiokanzoto was prepared at final concentrations of 0, 0.5, and 1.0 mg/mL, and the inhibitory effects on ATPase activity in the presence of 50 *μ*M each of the four P-gp substrates (verapamil, digoxin, vinblastine, and ketoconazole) were examined [14]. The final concentration of dimethylsulfoxide was <1%, at which no effect on the experimental system was found.

### 2.5. Inhibitory Effects of Daiokanzoto and Its Herbal Ingredients on ATPase Activity

Inhibitory effects of the herbal ingredients of Daiokanzoto on ATPase activity were studied in the presence of 50 *μ*M verapamil. The final concentrations of the herbal ingredients (rhubarb and licorice root) were 0.0625, 0.25, and 0.5 mg/mL. 

### 2.6. Concentration Dependence of Inhibitory Effects on ATPase Activity

Four concentrations of Daiokanzoto (0, 0.0625, 0.25, and 0.5 mg/mL) and seven concentrations of verapamil (0, 1.56, 3.13, 6.25, 12.5, 25, and 50 *μ*M) were tested, and the inhibitory effects of Daiokanzoto on ATPase activity in the presence of verapamil were investigated. The following analysis was performed after correction by subtracting from all values the ATPase activity at 0.5 mg/mL Daiokanzoto and 0 *μ*M verapamil. To examine inhibitory patterns, a Lineweaver-Burk plot was prepared using the reciprocal of ATPase activity (1/*V*) as the ordinate and the reciprocal of verapamil concentration (1/[*S*]) as the abscissa; linear regression was performed for each concentration of Daiokanzoto. A Dixon plot was prepared with the reciprocal of ATPase activity (1/*V*) as the ordinate and the reciprocal of the Daiokanzoto concentration as the abscissa. Linear regressions were performed for each concentration of verapamil, and Ki values were calculated from the values of the *x* coordinates at the intersection points on the graphs.

Rhubarb was investigated in the same manner as Daiokanzoto using concentrations of 0, 0.0625, 0.25, and 0.5 mg/mL.

### 2.7. Inhibitory Effects of Components of Rhubarb on ATPase Activity

The inhibitory effects of the 13 components of rhubarb (sennoside A, sennoside B, rhein, aloe emodin, emodin, gallic acid, (+)-catechin, (−)-catechin, (−)-catechin gallate, (−)-epicatechin, (−)-epicatechin gallate, (−)-epigallocatechin, and (−)-epigallocatechin gallate) on ATPase activity were studied in the presence of 50 *μ*M verapamil. The final concentrations of sennoside A, sennoside B, rhein, aloe emodin, emodin, and gallic acid were 0.002, 0.01, and 0.05 mg/mL. The final concentrations of (+)-catechin, (−)-catechin, (−)-catechin gallate, (−)-epicatechin, (−)-epicatechin gallate, (−)-epigallocatechin, and (−)-epigallocatechin gallate were 8, 40, and 200 *μ*M.

### 2.8. Statistical Analyses

The results are shown as the means ± SD, and statistical significance was evaluated via Student's *t*-test.

## 3. Results

### 3.1. Inhibitory Effects of Daiokanzoto on P-gp

**Figure 1:**
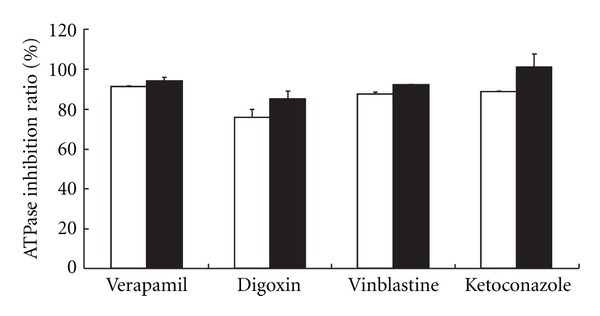
Inhibitory effects of Daiokanzoto on ATPase activity in the presence of various P-gp substrates. Daiokanzoto was added to a final concentration of 0.5 mg/mL (open column) or 1.0 mg/mL (closed column) together with verapamil, digoxin, vinblastine, or ketoconazole as the P-gp substrate (50 *μ*M). Data represent the mean ± SD of three experiments.

This study investigates the effects of Daiokanzoto on ATPase activity in the presence of four P-gp substrates (50 *μ*M each; verapamil, digoxin, vinblastine, and ketoconazole) [14]. Control values corresponded to the ATPase activities obtained when each substrate was added alone. Daiokanzoto showed significant inhibition of ATPase activity in the presence of all substrates (Figure 1).

**Figure 2:**
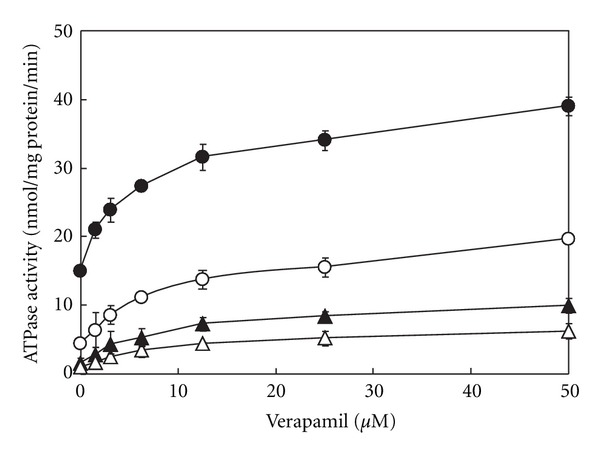
Inhibitory effects of Daiokanzoto on ATPase activity in the presence of verapamil. Daiokanzoto was added to a final concentration of 0 (•), 0.0625 (○), 0.25 (▲), or 0.5 (Δ) mg/mL together with various concentrations of verapamil. Data represent the mean ± SD of three experiments.

Among the P-gp substrates, verapamil resulted in the strongest inhibition of ATPase activity by Daiokanzoto. Therefore, the inhibitory effects of Daiokanzoto on ATPase activity in the presence of various concentrations of verapamil were studied. Daiokanzoto resulted in a concentration-dependent inhibition of ATPase activity at all concentrations of verapamil (Figure 2).

**Figure 3:**
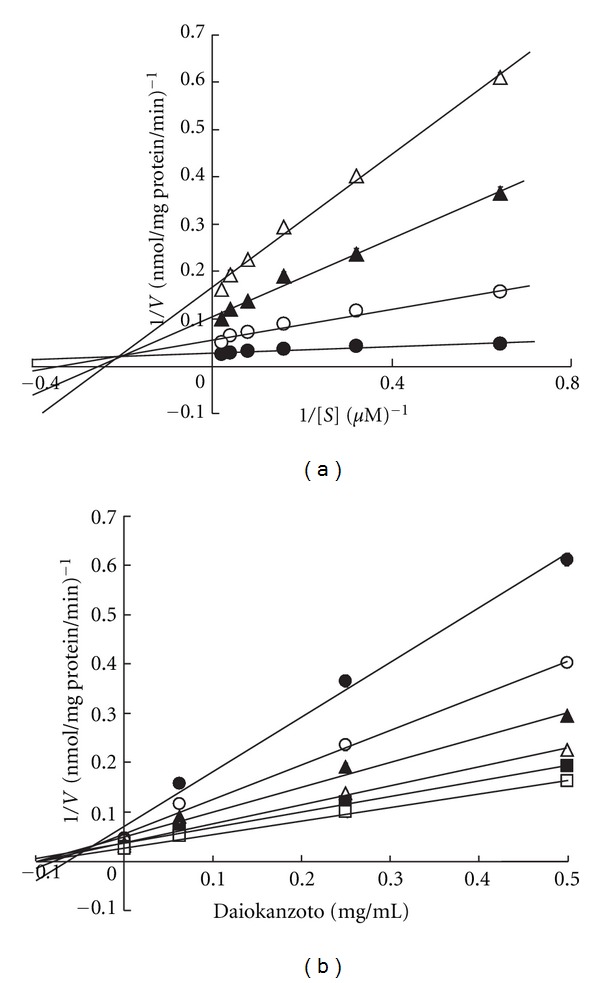
Lineweaver-Burk (a) and Dixon (b) plots for the inhibitory effects of Daiokanzoto on ATPase activity in the presence of verapamil. (a) The final concentration of Daiokanzoto was 0 (●), 0.0625 (○), 0.25 (▲), or 0.5 (Δ) mg/mL. (b) The final concentration of verapamil was 1.56 (●), 3.13 (○), 6.25 (▲), 12.5 (Δ), 25 (■), or 50 (□) *μ*M. Data represent the mean ± SD of three experiments. *V*: ATPase activity (nmol/mg protein/min), *S*: verapamil concentration (*μ*M).

The resulting inhibition patterns were investigated using the Lineweaver-Burk and Dixon plots. When linear regression was performed at each concentration of Daiokanzoto, the regression curves for both plots showed intersection points in the second quadrant (Figure 3). These results suggest that the P-gp inhibition pattern of Daiokanzoto in the presence of verapamil is a mix of competitive and noncompetitive inhibition. The Ki for Daiokanzoto, calculated from the *x* coordinate of the intersection point, was 0.057 mg/mL.

### 3.2. Inhibitory Effects of Rhubarb on P-gp

**Figure 4:**
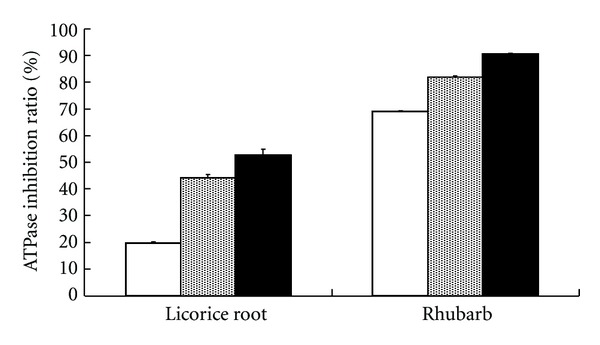
Inhibitory effects of rhubarb and licorice root on ATPase activity in the presence of verapamil.  Licorice root or rhubarb was added to a final concentration of 0.0625 (open column), 0.5 (dotted column), or 1 (closed column) mg/mL together with 50 *μ*M verapamil. Data represent the mean ± SD of three experiments.

Because Daiokanzoto was found to have concentration-dependent inhibitory effects on ATPase activity, the inhibitory effects of the herbal ingredients, rhubarb and licorice root, were examined to determine which herbal ingredient caused these inhibitory effects. Both rhubarb and licorice root (0.0625, 0.25, and 0.5 mg/mL) significantly inhibited ATPase activity in the presence of 50 *μ*M verapamil (Figure 4).

**Figure 5:**
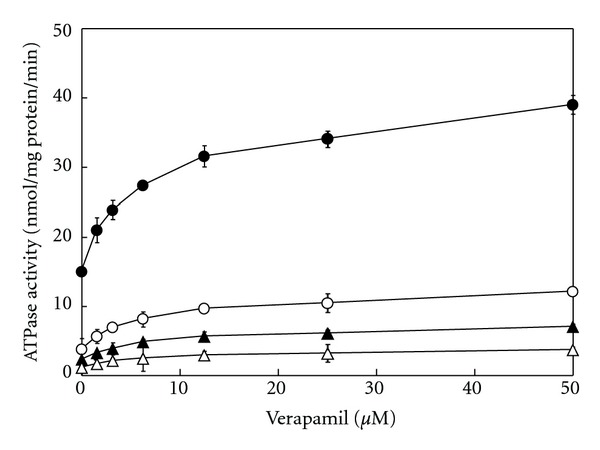
Inhibitory effects of rhubarb on ATPase activity in the presence of verapamil. Rhubarb was added to a final concentration of 0 (●), 0.0625 (○), 0.25 (▲), or 0.5 (Δ) mg/mL together with various concentrations of verapamil. Data represent the mean ± SD of three experiments.

The inhibitory effects of rhubarb on ATPase activity in the presence of verapamil at various concentrations were studied. Rhubarb demonstrated a concentration-dependent inhibition of ATPase activity at all concentrations of verapamil studied (Figure 5).

**Figure 6:**
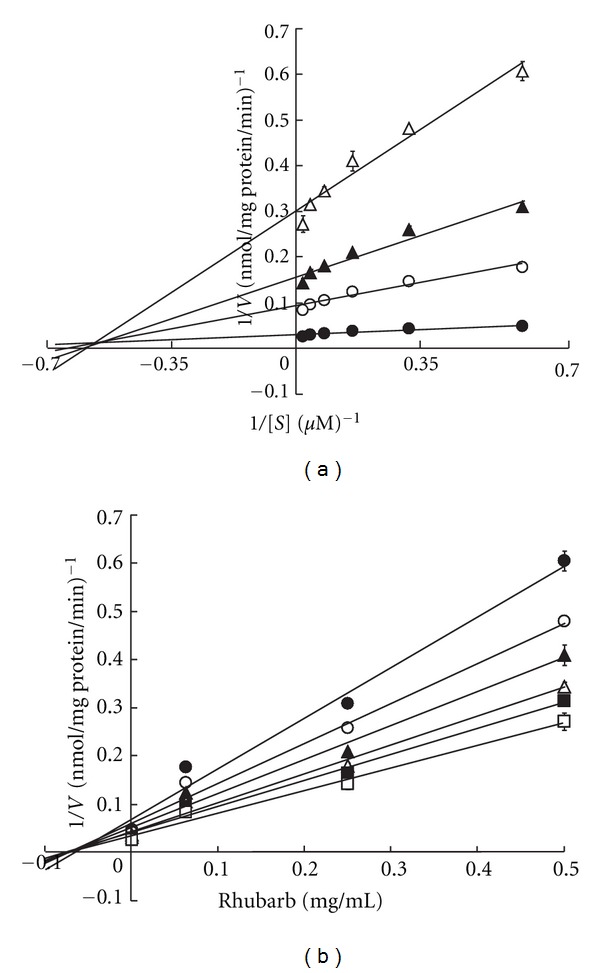
Lineweaver-Burk (a) and Dixon (b) plots for the inhibitory effects of rhubarb on ATPase activity in the presence of verapamil. (a) The final concentration of rhubarb was 0 (●), 0.0625 (○), 0.25 (▲), or 0.5 (Δ) mg/mL. (B) The final concentration of verapamil was 1.56 (●), 3.13 (○), 6.25 (▲), 12.5 (Δ), 25 (■), or 50 (□) *μ*M. Data represent the mean ± SD of three experiments. *V*: ATPase activity (nmol/mg protein/min), *S*: verapamil concentration (*μ*M).

The resulting inhibition patterns were investigated using Lineweaver-Burk and Dixon plots. Linear regressions were performed at each concentration of rhubarb, and the regression curves of both plots contained intersections in the second quadrant. These results suggest that the P-gp inhibition pattern of rhubarb and verapamil is a mix of competitive inhibition and noncompetitive inhibition similar to that observed for Daiokanzoto (Figure 6). The Ki of rhubarb (calculated as the *x* coordinate of the intersection point) was 0.063 mg/mL.

### 3.3. Effects of Components of Rhubarb on ATPase Activity

**Figure 7:**
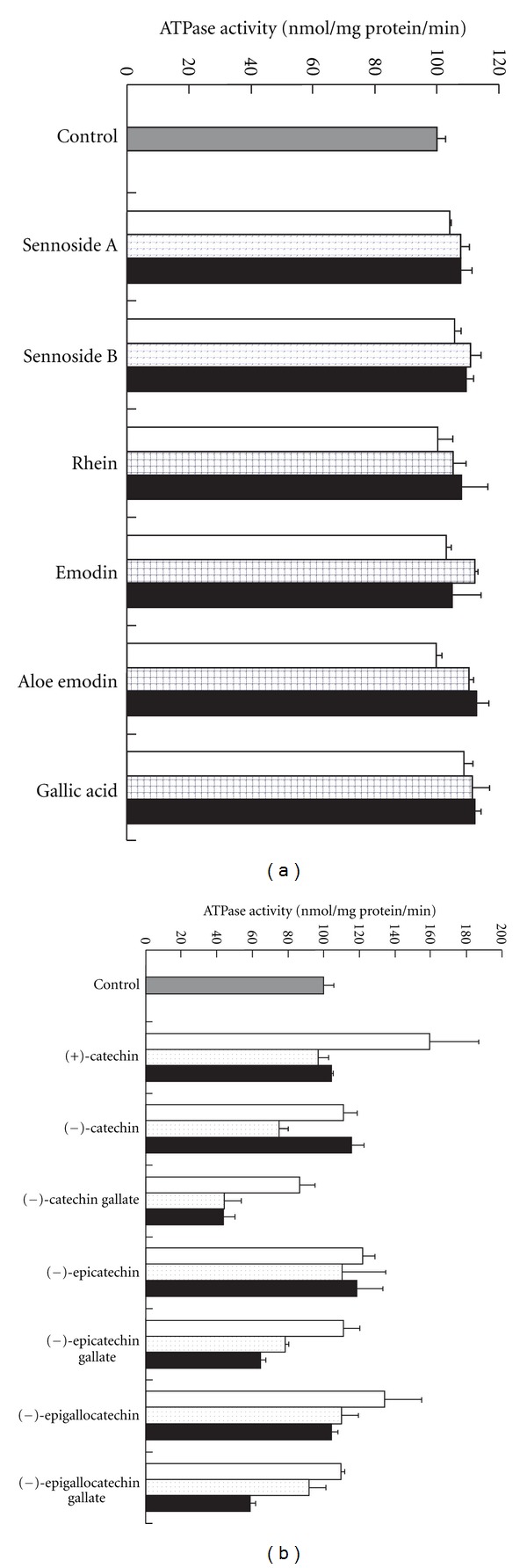
Effects of the components of rhubarb on ATPase activity in the presence of verapamil. (a): Each component was added to a final concentration of 0.002 (open column), 0.01 (dotted column), or 0.05 (closed column) mg/mL together with 50 *μ*M verapamil. B: Each component was added to a final concentration of 8 (open column), 40 (dotted column), or 200 (closed column) *μ*M together with 50 *μ*M verapamil. Data represent the mean ± SD of three experiments.

The inhibitory effects of the 13 known components of rhubarb on ATPase activity were studied in the presence of 50 *μ*M verapamil. Sennoside A, sennoside B, rhein, aloe emodin, emodin, gallic acid, (+)-catechin, (−)-catechin, (−)-epicatechin, and (−)-epigallocatechin did not show significant effects on ATPase activity in the presence of verapamil (Figure 7). In contrast, (−)-catechin gallate, (−)-epicatechin gallate, and (−)-epigallocatechin gallate significantly inhibited ATPase activity in a concentration-dependent manner.

## 4. Discussion

The present study investigates the inhibitory effects of Daiokanzoto and its herbal ingredients on P-gp via an ATPase assay using human P-gp membranes. The reaction solution in ATPase assay contained EGTA, ouabain, and sodium azide, which inhibits Ca^2+^-ATPase, Na^+^, K^+^-ATPase, and mitochondrial ATPase, respectively. Therefore, the inhibitory activity is specific for P-gp. In addition, this ATPase assay has been reported to enable more efficient screening for substrates and inhibitors of P-gp compared with other *in vitro* assays (such as the transcellular transport assay) [18].

Daiokanzoto showed significant inhibitory effects on ATPase activity in the presence of all P-gp substrates used (Figure 1). A kinetic analysis of the inhibitory effects on ATPase activity in the presence of verapamil resulted in regression curves that, for both the Lineweaver-Burk and Dixon plots, contained intersection points in the second quadrants for all concentrations studied (Figure 3). Therefore, the inhibition pattern of Daiokanzoto for verapamil represents a mix of competitive and noncompetitive inhibition processes.

The present study regarding the inhibitory effects of rhubarb and licorice root (as herbal ingredients of Daiokanzoto) on ATPase activity in the presence of verapamil reveals that both components exert inhibitory effects, with rhubarb being the stronger of the two (Figure 4). Comparing these results with those in Figure 1 and acknowledging the fact that Daiokanzoto contains twice the amount of rhubarb as licorice root, the results of this study suggest that rhubarb is likely the primary contributor to the inhibitory effects of Daiokanzoto. 

Rhubarb showed concentration-dependent inhibition of ATPase activity in the presence of verapamil (Figure 5). When kinetic analysis was conducted on these inhibitory effects, both Lineweaver-Burk and Dixon plots showed the same patterns as those obtained for Daiokanzoto (Figure 6), indicating that the inhibition pattern of rhubarb is also a mix of competitive and noncompetitive inhibition(s). Given the same inhibition patterns obtained for Daiokanzoto and rhubarb and the fact that the Ki value of rhubarb was estimated to be 11% larger than that of Daiokanzoto, this study suggests that the inhibitory effects of Daiokanzoto are primarily caused by rhubarb.

Thirteen components of rhubarb were studied to clarify which are responsible for the inhibitory effects of rhubarb on ATPase. Of these, sennoside A, sennoside B, rhein, aloe emodin, emodin, gallic acid, (+)-catechin, (−)-catechin, (−)-epicatechin, and (−)-epigallocatechin were found to exhibit no inhibitory effects on ATPase activity even at sufficiently high concentrations, whereas (−)-catechin gallate, (−)-epicatechin gallate, and (−)-epigallocatechin gallate demonstrated concentration-dependent inhibitory effects (Figure 7). A study regarding the uptake of a fluorescent substrate by NIH-3T3-G185 cells transfected with the human MDR1 gene showed similar P-gp inhibitory effects [19]. However, to cause the inhibitory effects of rhubarb observed as a result of the presence of (−)-catechin gallate, (−)-epicatechin gallate, and (−)-epigallocatechin gallate, these compounds would have to compose nearly 100% of rhubarb. The content of (−)-epicatechin gallate in rhubarb is reportedly less than 0.1% [20]; thus, the observed inhibition cannot be explained by (−)-epicatechin gallate. Intestinal bacteria almost completely hydrolyze (−)-epicatechin gallate and (−)-epigallocatechin gallate to form (−)-epicatechin and (−)-epigallocatechin, which are absorbed [21]. Thus, these substances are unlikely to reach the physiological concentrations required for *in vivo* inhibition.

The present results indicate that rhubarb may cause changes in the disposition of P-gp substrates via the inhibition of P-gp. It is known that P-pg-expressing cancer cells are resistant to anticancer drugs. Therefore, rhubarb may potentiate the anticancer effect by the inhibition of P-gp. In the future, it is worthwhile to study interactions with Kampo medicines containing rhubarb as a herbal ingredient.
